# A perspective on the early days of *RAS* research

**DOI:** 10.1007/s10555-020-09919-1

**Published:** 2020-07-29

**Authors:** Robin A. Weiss

**Affiliations:** Division of Infection & Immunity, University College London, Gower Street, London, WC1E 6BT UK

**Keywords:** Ras, Retrovirus, Oncogene, DNA transfection, Signal transduction, Chris Marshall, Alan Hall

## Abstract

The name of the oncogene, *ras*, has its origin in studies of murine leukemia viruses in the 1960s by Jenny Harvey (H-*ras*) and by Werner Kirsten (K-*ras*) which, at high doses, produced sarcomas in rats. Transforming retroviruses were isolated, and its oncogene was named *ras* after rat sarcoma. From 1979, cellular *ras* sequences with transforming properties were identified by transfection of tumor DNA initially by Robert Weinberg from rodent tumors, and the isolation of homologous oncogenes from human tumors soon followed, including *HRAS* and *KRAS*, and a new member of the family named *NRAS*. I review these discoveries, placing emphasis on the pioneering research of Christopher Marshall and Alan Hall, who subsequently made immense contributions to our understanding of the functions of *RAS* and related small GTPases to signal transduction pathways, cell structure, and the behavior of normal and malignant cells.

## Introduction

For this special issue on *Mutant KRAS: Hidden Secrets in Tumor Progression*, the Guest Editor, Jozsef Timar, asked me to write a retrospective piece on the important contributions to *RAS* research by my protégés, Chris Marshall and Alan Hall, who have sadly predeceased me. I accepted this kind invitation, feeling pleased to honor the names of these two innovative and talented scientists. I have, however, included a broader perspective on the contribution of retroviruses and of DNA transfer techniques to the development of molecular oncology for the benefit of readers who may not be well versed in the history of the field and to provide a context to early *RAS* research.

On the somewhat confusing issue of nomenclature of oncogenes, it is customary to refer to genes in italics and proteins non-italicized with a capital first letter. Rodent oncogenes are denoted in the lower case, such as Ha-*ras*, whereas human oncogenes have three capital letters and no hyphen, such as *KRAS*. Rodent *ras* genes used to have prefixes, Ha- for Harvey *ras* and Ki- for Kirsten *ras*, whereas human genes drop the second letter. Oncogenes in retroviruses are denoted as viral v-*onc* and their progenitors in host DNA as cellular c-*onc*.

## Discovery of oncogenes including *ras* in retroviruses

Oncogenic retroviruses have a long story line going back to the isolation of filterable agents (not yet called viruses) in domestic fowl (chickens) by the Danish veterinarians Ellerman and Bang in 1908 who recorded transmission of erythroleukemia and by Peyton Rous who discovered the sarcoma agent which now bears his name, Rous sarcoma virus (RSV). But it was not until Temin and Rubin devised a quantitative assay *in vitro* for RSV in 1958 that experimental cell and molecular biology of transforming genes took flight [1].

The transformation of cells in culture by RSV was found to be due to the prototypic oncogene, *src*, which, while not contributing to viral replication, proved to be essential for cell transformation. Moreover, all oncogene-bearing retroviruses, except for certain European strains of RSV, are defective for virus replication because the oncogene has substituted for or fused with an essential gene for replication, and they therefore depend on the presence of replication-competent leukemia virus for complementation of the missing functions. The *src* oncogene was transduced and modified from a cellular gene. Many other oncogenes of cellular origin also first came to light in oncogenic retroviruses of chickens and mammals, for example, *abl*, *myc*, and, of course, *ras* [1, 2].

The first retrovirus containing a *ras* oncogene was reported by Jenny Harvey in London in 1964 [3]. A related virus was isolated by Kirsten and Mayer in Chicago in 1967 [4] which contained a related but distinct *ras* oncogene. They called the transforming viruses as murine sarcoma virus (MSV), and they were derived by infecting rats with high doses of murine leukemia viruses (MLV). Later, a virus closely related to Ha-MSV was obtained by Rasheed et al. by passage of MLV in rat cells *in vitro* [5].

Curiously, sarcoma viruses containing *ras* have not been isolated from mice or from other mammals. Mice have generated sarcoma viruses from MLV, but they contain other oncogenes, such as *fos* in the FBJ-MSV inducing osteosarcoma [6]. Cats infected with feline leukemia virus frequently give rise to sarcoma viruses [7], but they contain *fes* or *fms* oncogenes. Thus, the transduction of *ras* exclusively from rat cells infected by MLV remains a conundrum. Ki-MSV evolved by mutation and complex recombination events of c-*ras* in rats with endogenous retroviral elements in rats and with the exogenous MLV [8].

## Discovery of cellular *ras* genes through DNA transfection

The transfer of genetic material from one cell to another was pioneered by the British bacteriologist, Fred Griffith, in 1928 [9]. He detected a “Transforming Principle” in extracts of a heat-inactivated virulent “S” strain of *Pneumoccocus* that conferred its virulence to a non-virulent “R” strain. This transfection technique led to one of the most important discoveries of the mid-twentieth century by Oswald Avery, Colin MacLeod, and Maclyn McCarty in 1944 [10], namely, that DNA is the genetic material, rather than protein as previously assumed. Without this breakthrough by Avery et al. [10], Maurice Wilkins, Rosalind Franklin, James Watson, and Francis Crick would not have studied the structure of DNA and discovered the double helix.

The transfection technique was also applied to oncogenic viruses. In 1936, George Berry and Helen Dedrick transferred a virulence marker from inactivated myxoma pox virus to the related benign Shope fibroma [11]. In 1971, the year after the discovery of reverse transcriptase, Miroslav Hill and Jana Hillova reported the rescue of infectious RSV from RSV-transformed rat tumor cells using DNA transfection, published initially in French and subsequently in English [12]. The rat tumor cells, XC, had been established many years earlier by their mentor, Jan Svoboda; although XC cells were not permissive for RSV replication, Svoboda had shown in several studies that they contained a replication-competent form of RSV carrying the *src* oncogene because it could be rescued by fusing the rat cells with permissive chick embryo fibroblasts, which I have reviewed elsewhere [13]. Hill and Hillova’s experiment represented the formal validation of Howard Temin’s DNA provirus hypothesis, and it was also the first demonstration of reliable DNA transfection of an oncogene [12].

DNA transfection methods became more efficient with the use of calcium phosphate for transfer of the human adenovirus type 5 genome [14] and the use of murine NIH-3T3 cells [15] as target cells. NIH-3T3 cells proved to be very useful for oncogene studies because they grow as an immortal cell line with high cloning efficiency but without a morphologically transformed phenotype. After Michael Bishop, Harold Varmus, and colleagues [16] showed that retroviral oncogenes were derived from cellular proto-oncogenes, it seemed logical to attempt to transfer cellular DNA from nonviral tumors in order to detect transforming genes. The breakthrough was achieved by Robert Weinberg and colleagues [17] using murine tumors induced by chemical carcinogens.

Weinberg’s success [17] led to an explosion of studies of oncogenes by DNA transfection over the next 5 years, as reviewed in detail in 1985 by Chris Marshall [18] and later by Malumbres and Barbacid [19]. In addition to morphological transformation of NIH-3T3 cells, the transforming activity of tumor DNA could also be detected by growth of cells in soft agar suspension [20] and as tumors in immunodeficient mice [21]. It was soon found that some human tumor cells lines yielded transfectable oncogenes, the first being the human bladder carcinoma cell line EJ [22, 23]. Oncogenes were then detected in numerous types of human tumor, including colorectal and lung carcinomas, melanoma, neuroblastoma, sarcoma, and hematologic tumors [18].

The identification of *RAS* as the oncogene in many of these human tumors was first found with the EJ bladder cell line since sequences homologous to Ha-*ras* of MSV were detected by DNA hybridization and cloning [24, 25]. The majority of oncogenes from colon and lung carcinomas were homologs of Ki-*ras* of MSV [18]. At the time, I was mildly surprised that most of the newly identified human oncogenes turned out to be “old friends” from retrovirus research.

## Interplay of tumor suppressor genes and oncogenes

The concept of tumor suppressor functions came from three sources. The first was the analysis pioneered in 1971 by Alfred Knudson [26] based on the familial susceptibility of children to retinoblastoma and to other tumors if retinoblastoma was cured. His “2-hit” model was that a dominant gene, *Rb*1, prevents cell transformation and that susceptible children are germ-line heterozygotes for *Rb*1, carrying one functional wild-type allele and a defective allele. Then, if one of the millions of retinoblasts developed a somatic mutation in the wild-type allele, no functional Rb1 protein would be expressed, and a clonal tumor developed from that mutant cell. This is analogous to activation of *KRAS* by somatic mutation except that it represents a loss of function rather than a gain in function.

The second line of evidence came from the analyses of somatic cell hybrids between normal and malignant cells in which the normal phenotype tends to dominant, pioneered by Henry Harris and his group in Oxford [27], which in the early 1970s included a doctoral student, Christopher Marshall. Hybrids between murine tumor cells and normal human cells proved to be informative because, when the hybrids spontaneously lost human chromosomes upon cell passage, they reverted to a transformed phenotype, and thus the location of tumor suppressor genes could be mapped to specific chromosomes.

The third discovery was of p53 protein through research on the oncogenic simian virus 40 (SV40) in 1979—the same year as Weinberg’s first transfection of Ha-*ras* from murine tumors [17]. It was observed by David Lane and Lionel Crawford [28], and by Daniel Linzer and Arnie Levine [29], that immunoprecipitation of the oncogene product of SV40, large T (tumor) antigen, co-precipitated a cellular protein of 53,000–54,000 Da, now known as TSp53. The TSp53 protein was not merely a contaminant of the precipitates and gels, but was specifically bound to large T. It was found that large T blocks TSp53 function, just as somatic mutations in TSp53, can contribute to malignant transformation, thus preventing TSp53 to act as what Lane dubbed the “Guardian of the genome” [30].

It is remarkable that so many oncogenic DNA viruses target TSp53 (and also Rb1). Not only does polyoma large T abrogate TSp53 function but also E1B of adenovirus, E6 of cervical human papilloma viruses and latent nuclear antigen of Kaposi’s sarcoma virus. These viral proteins are unrelated to each other in sequence and structure, yet, by blocking TSp53, they serve in common to initiate the S phase of the host cell mitotic cycle to permit viral DNA replication. The rare cell that proceeds to clonal expansion to form a tumor may be regarded as collateral damage [31].

## Contributions by Christopher Marshall and Alan Hall

In 1979, I was appointed Director-designate of the Institute of Cancer Research (ICR), Royal Marsden Hospital, London, where there was space to establish new research groups in the Chester Beatty Laboratories. The Institute was strong in several areas and had access to human biopsies from the hospital, but it lacked modern cell and molecular biology approaches to the cancer problem. Seeing that the newly discovered cellular oncogenes and tumor suppressor genes were ripe for exploitation, I sought to recruit scientists who could apply these areas to human tumors.

Chris Marshall already had established a reputation in somatic cell genetics in relation to suppression of the transformed phenotype of tumors after fusion with non-malignant cells [32]. He joined the ICR from the Dana-Farber Center at Harvard shortly after my arrival in May 1980, with a grant to extend his studies of tumor suppression. Meanwhile, I gained a grant to search for oncogenes in human tumors by DNA transfection, which had not yet been reported by other research groups. We needed to acquire the then novel method of recombinant DNA technology, and we were fortunate to recruit Alan Hall supported on my grant; he was a molecular biologist who had participated in cloning the interferon alpha gene in Charles Weissmann’s laboratory in Zurich [33].

### Discovery of *NRAS*

As it turned out, Chris and Alan immediately got along well together. Rather than pursue tumor suppressor genes, Chris decided to join our effort to search for human oncogenes. Over the course of many months, they tested DNA samples transfected from various human tumors and cell lines without observing NIH-3T3 cell transformation. They were about to give up when the first reports of successful transfection with human EJ bladder tumor cell line were published [22, 23]. Encouraged by the success of the other groups, they decided to try once more and obtained hits with DNA from two human sarcoma cell lines, the HT1080 fibrosarcoma and the RD pediatric rhabdomysarcoma. DNA hybridization analysis indicated that the transforming gene was the same in both sarcomas, but it differed from sequences reported by others in human tumors and had no obvious homolog among retrovirus oncogenes [34].

Chris and Alan set out to characterize the new sarcoma oncogene by molecular cloning. Meanwhile, Michael Wigler’s group isolated the same oncogene from the neuroblastoma cell line, SK-N-SH [35], and it was also later identified in HL60 promyeolcytic leukemia cells. Hall and Marshall found that the gene was a hitherto unknown member of the *RAS* family, more distantly related than *HRAS* and *KRAS* are to each other [36]. It became known as *NRAS*, the *N* standing for neuroblastoma or “new.” *NRAS* is located on human chromosome 1 on the centric portion of the p arm [36, 37], while *HRAS* and *KRAS* are located on human chromosomes 11 and 12, respectively. It is noteworthy that *RAS* represents a family of genes, whereas most types of oncogene have a single member. Since *NRAS* is expressed in sarcomas, neuroblastoma, and several types of leukemia and *HRAS* and *KRAS* in many types of carcinoma and leukemia, the biological reason for the diversification of *RAS* genes is unclear.

### *RAS* function and activation

Before the discovery of human *RAS* genes, it was already known that the retroviral v-Ha-*ras* and v-Ki-*ras* oncogenes encode the p21 proteins that act as small GTPases which transduce cell growth and survival signals. Robin Brown in Chris and Alan’s laboratory showed that *NRAS* in HT1080 sarcoma cells has an activating mutation in codon 61 [38]. Somatic mutations, particularly in and around amino-acid residues 12 and 61, prevent degradation by GTP hydrolysis, and therefore, RAS becomes locked into a constitutively active signaling state [39, 40].

Chris and Alan were so successful in pursuing the intricacies of human *RAS* that I realized my role in the project was superfluous, other than providing financial and occasional intellectual support. Within 2 years of his arrival at ICR, Alan had won a research grant and established his own independent team, alongside Chris. I therefore bowed out of *RAS* research and followed my interest in the interface between cancer and infection. With the discovery in the early 1980s of the human retroviruses, human T cell leukemia virus, and human immunodeficiency virus, I turned my attention to them, later adding Kaposi’s sarcoma herpesvirus and contagious tumors for which the transmissible agent is the tumor cell itself.

After their success in identifying *NRAS*, Chris and Alan switched to studying the complex downstream signaling pathways by which GTPase oncogenes and their normal counterparts control cell metabolism, cell proliferation, and cell motility and shape. These lines of inquiry remained their focus of research for the rest of their careers, and it is chiefly what they are remembered for today. The delineation of the RAS-MAP-kinase signaling cascade of kinases and phosphatases owes much to the in-depth research of Marshall’s laboratory [41]. He also demonstrated that post-translational prenylation of p21-Ras is necessary for its membrane attachment and signaling function [42] and went on to examine whether agents that block the addition of lipophilic farnesyl or geranyl-geranyl groups to RAS might act as cancer inhibitors. Recently, blocking prenylation has aroused renewed interest as a therapeutic target [43].

Alan Hall pioneered investigation of the role of Rac, Rho, and cdc42, (GTPases related to Ras) on the regulation of cell shape and movement in relation to metastasis. In this area, I was particularly taken with the work of Anne Ridley and Hall on focal adhesions, actin stress fibers, and membrane ruffling published consecutively in *Cell* [44, 45] because my doctoral studies in Michael Abercrombie’s laboratory 25 years earlier (when we lacked molecular and biochemical techniques) had concerned contact inhibition of cell locomotion and growth and its loss in RSV-transformed cells. The cell biologist and microscopist, Hugh Patterson, who was a long-term associate of Chris, aided these studies with microinjection and time-lapse microcinematography [46].

I shall not provide a detailed account or bibliography here of Marshall’s and Hall’s studies of the RAS family, because this article is focused on early research, and the other contributors to this issue provide current perspectives, especially to *KRAS*. Besides, the insights provided by their groups in the complex network of cell signaling at the molecular level is well-known [47] and has been beautifully reviewed by Marshall [41, 47, 48] and Hall [49–52] themselves.

Chris remained at the ICR throughout his career, where he led the Division of Cell and Molecular Biology and later became Research Director. He foresaw the application of the RAS signaling cascade as targets for cancer therapy. Alan and Chris worked closely together at ICR until Alan moved in 1993 to the Medical Research Council Laboratory of Molecular Cell Biology at University College London, where he became Director in 2000. In 2004, he moved to Memorial Sloan-Kettering Institute in New York as Chair of Cell Biology. They were both elected Fellows of the Royal Society (the British and Commonwealth premier Academy of Sciences) while still in their 40s, a remarkable achievement, and won several prestigious prizes. Equally important to their illustrious research reputations, to my mind, was their role as mentors of a string of talented students and postdoctoral scientists, too numerous to name individually here, who went on to make notable discoveries. They retained the loyalty of their protégés and the affection and respect of their competitors, through their intellectual honesty and generosity of spirit to the research community at large.

Chris and Alan remained firm friends until their untimely deaths in 2015. Chris had a reputation for making caustic comments yet he had a heart of gold; Alan was more reserved but had a wry sense of humor. In May 2015, while jogging in New York, Alan collapsed and died, a shock to everyone who knew him. Chris had been suffering from colorectal cancer for a number of years, and the best treatment available at the Royal Marsden Hospital ultimately could not save him. I last met Chris at Alan’s funeral when he delivered a magnificent eulogy. Sadly, 3 months later he also died. Their major accomplishments are described in several of the obituaries written by their colleagues and protégés [53–56].

**Fig. 1:**
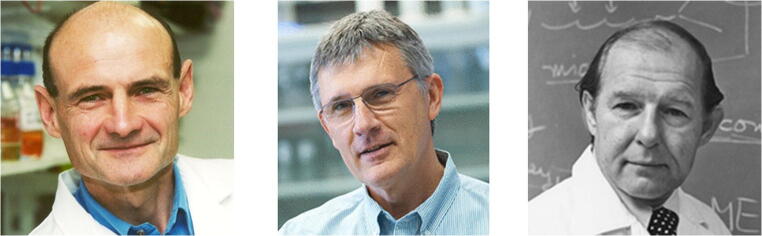
Left, Christopher J. Marshall, 1949–2015; Center, Alan Hall, 1952–2015; Right, Werner H. Kirsten, 1925–1992. Reproduced courtesy of the Institute of Cancer Research (CJM), Memorial Sloan-Kettering (AH), and the University of Chicago Photographic Archive [apf 7-00560], University of Chicago Library (WHK)

It is ironic that Chris was slain by a tumor on which he had contributed so much to our understanding of cell signaling and its deviations. I remember both Chris Marshall and Alan Hall with personal warmth as well as admiration for their research. I also fondly recall the superb pathologist and kind mentor of an earlier generation, Werner Kirsten, at the University of Chicago and later as Director of the NIH Frederick Cancer Research Facility, where the Werner H Kirsten (WHK) Student Intern Program still runs each summer. Kirsten’s biggest contribution to oncology is symbolized by the initial letter of his surname in *KRAS* (Fig. 1).
